# Supplementary material for: Synthesis of Novel Bromophenol with Diaryl Methanes—Determination of Their Inhibition Effects on Carbonic Anhydrase and Acetylcholinesterase
Source: Molecules. 2022 Nov 1;27(21):7426. doi: 10.3390/molecules27217426 (PMC9655968; doi:10.3390/molecules27217426)

Supplementary Material

Synthesis of Diaryl Methanes Like Novel Bromophenol and Their inhibition effect on Carbonic Anhydrase and Acetylcholinesterase Enzyme Activity

Necla ÖZTAŞKIN[a*], Süleyman GÖKSU*[a], Yeliz DEMİR[b],
Ahmet MARAŞ[a], İlhami GÜLÇİN[a]

[a]Department of Chemistry, Faculty of Science, Atatürk University, 25240-Erzurum-Turkey

[b]Department of Pharmacy Services, Göle Nihat Delibalta Vocational High School, Ardahan University, 75000-Ardahan, Turkey

^{1}H-NMR, ^{13}C-NMR and IR spectra

Table of Contents	Page (NMR)	Page (IR)
**1-bromo-2-(2-bromo-4-methoxybenzyl)-4,5-dimethoxybenzene(13)**	**S2**	**S11**
**1-bromo-4,5-dimethoxy-2-(5-methoxy-2-methylbenzyl)benzene (14)**	**S3**	**S11**
**1-bromo-2-(4-bromo-2,5-dimethoxybenzyl)-4,5-dimethoxybenzene (15)**	**S4**	**S12**
**1-bromo-2-(2,5-dimethoxybenzyl)-4,5-dimethoxybenzene (16)**	**S5**	**S12**
**1-bromo-2-(2,5-dimethoxybenzyl)-4,5-dimethoxybenzene (17)**	**S6**	**S13**
**4-bromo-5-(2-bromo-4-hydroxybenzyl)benzene-1,2-diol (18)**	**S7**	**S13**
**4-bromo-5-(2-bromo-4-hydroxybenzyl)benzene-1,2-diol (19)**	**S8**	**S14**
**4-bromo-5-(4-bromo-2,5-dihydroxybenzyl)benzene-1,2-diol (20)**	**S9**	**S14**
**4-bromo-5-(2,5-dihydroxybenzyl)benzene-1,2-diol (21)**	**S10**	**S15**

^{1}H-NMR and ^{13}C-NMR of compound 13($CDCl_3$)

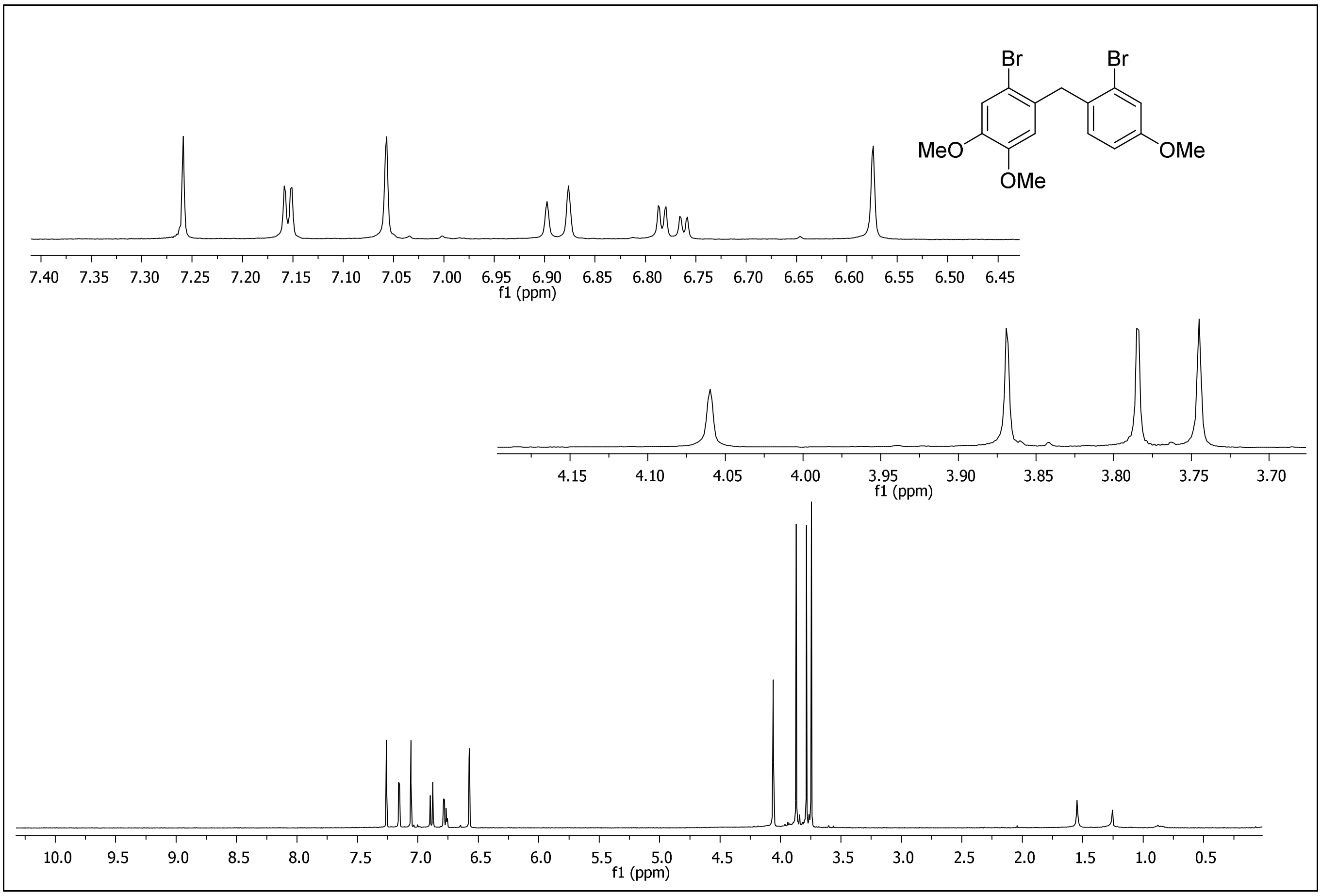

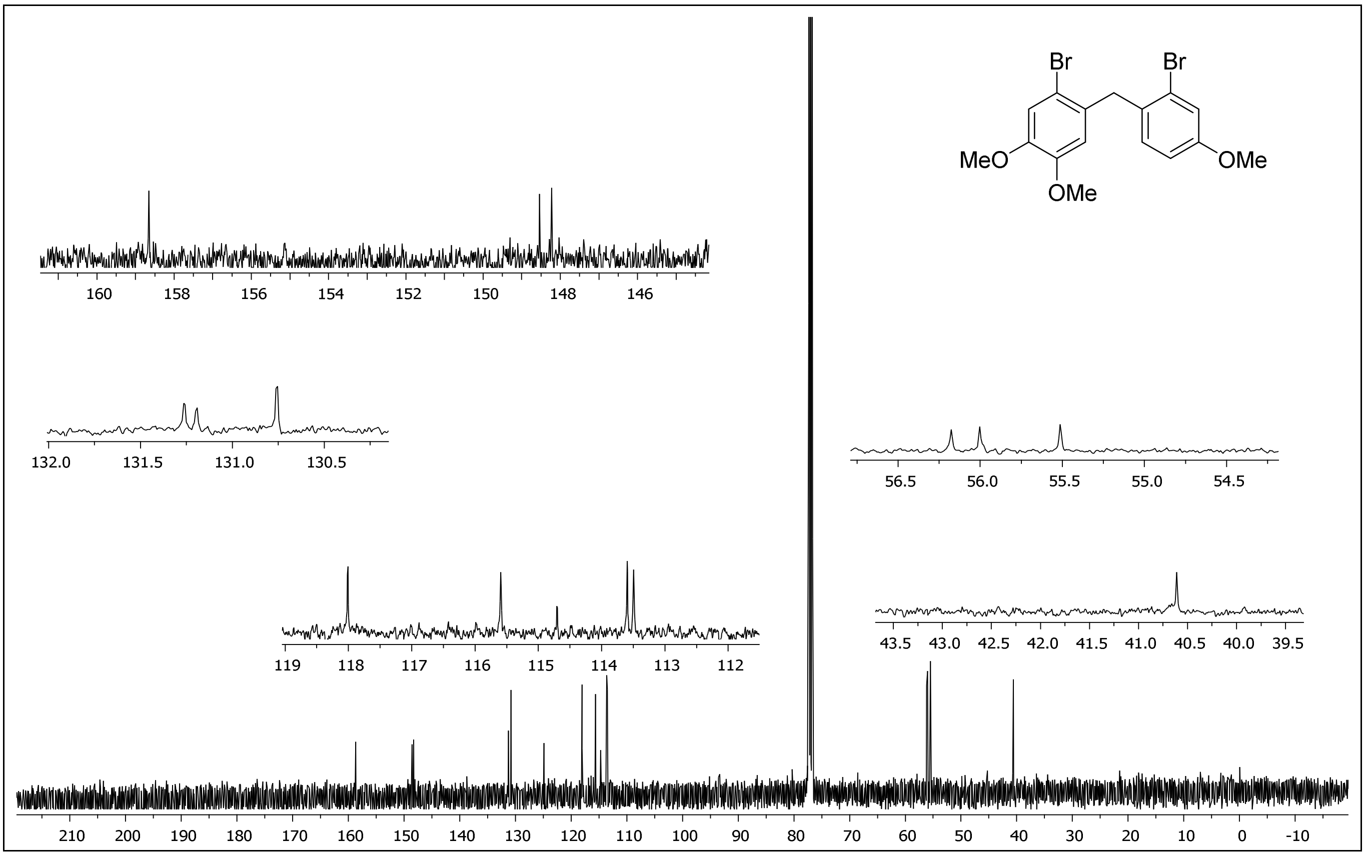

^{1}H-NMR and ^{13}C-NMR of compound 14 ($CDCl_3$)

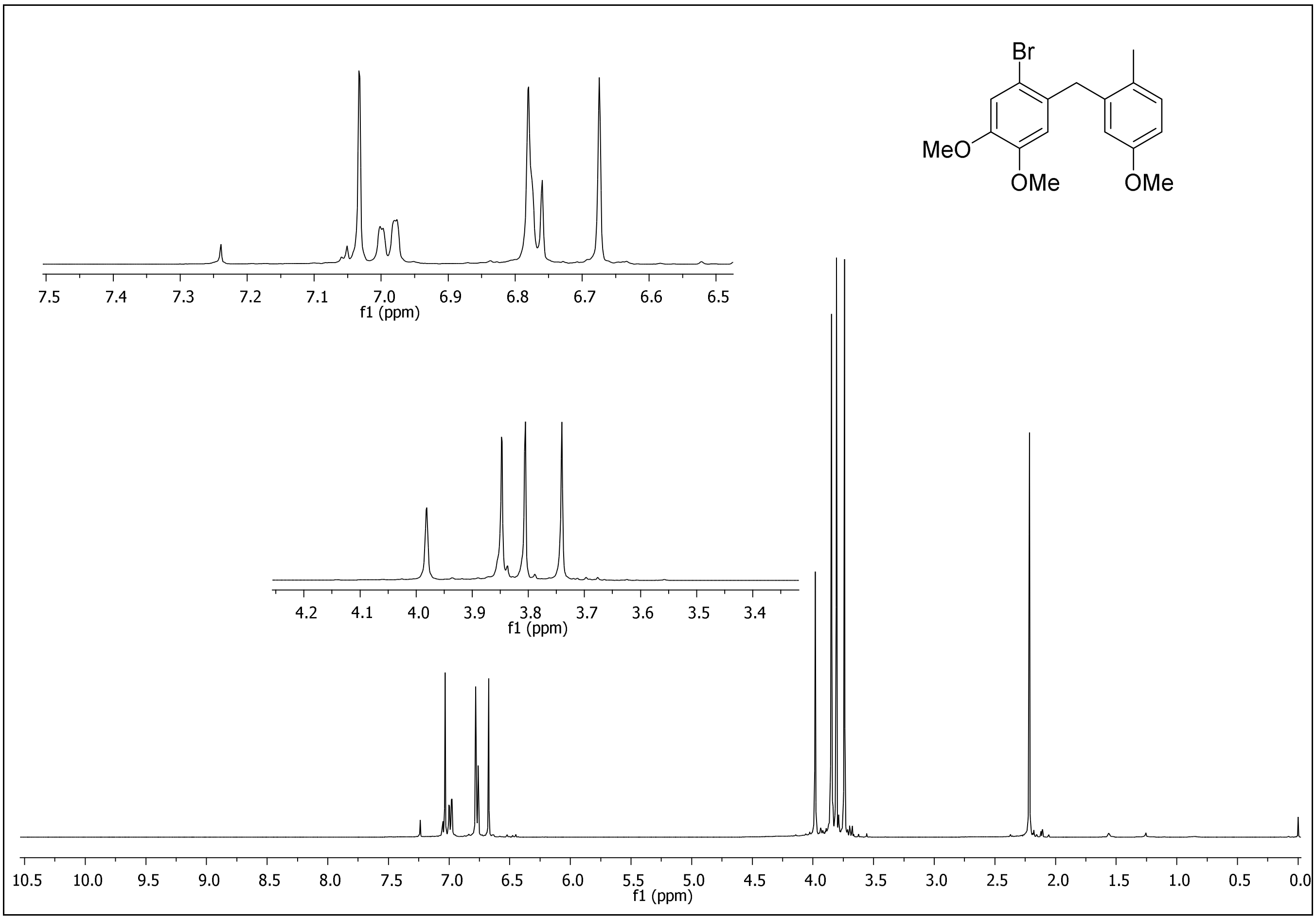

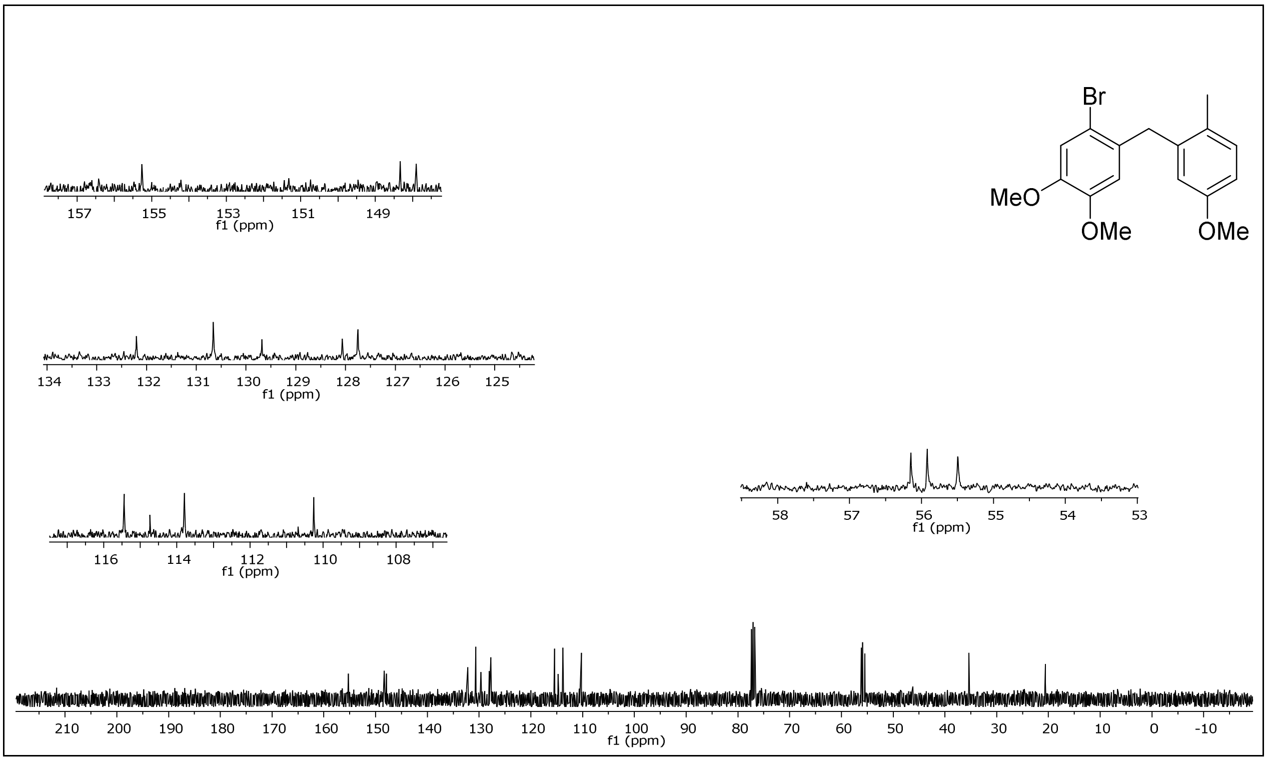

^{1}H-NMR and ^{13}C-NMR of compound 15(CDCl$_3$)

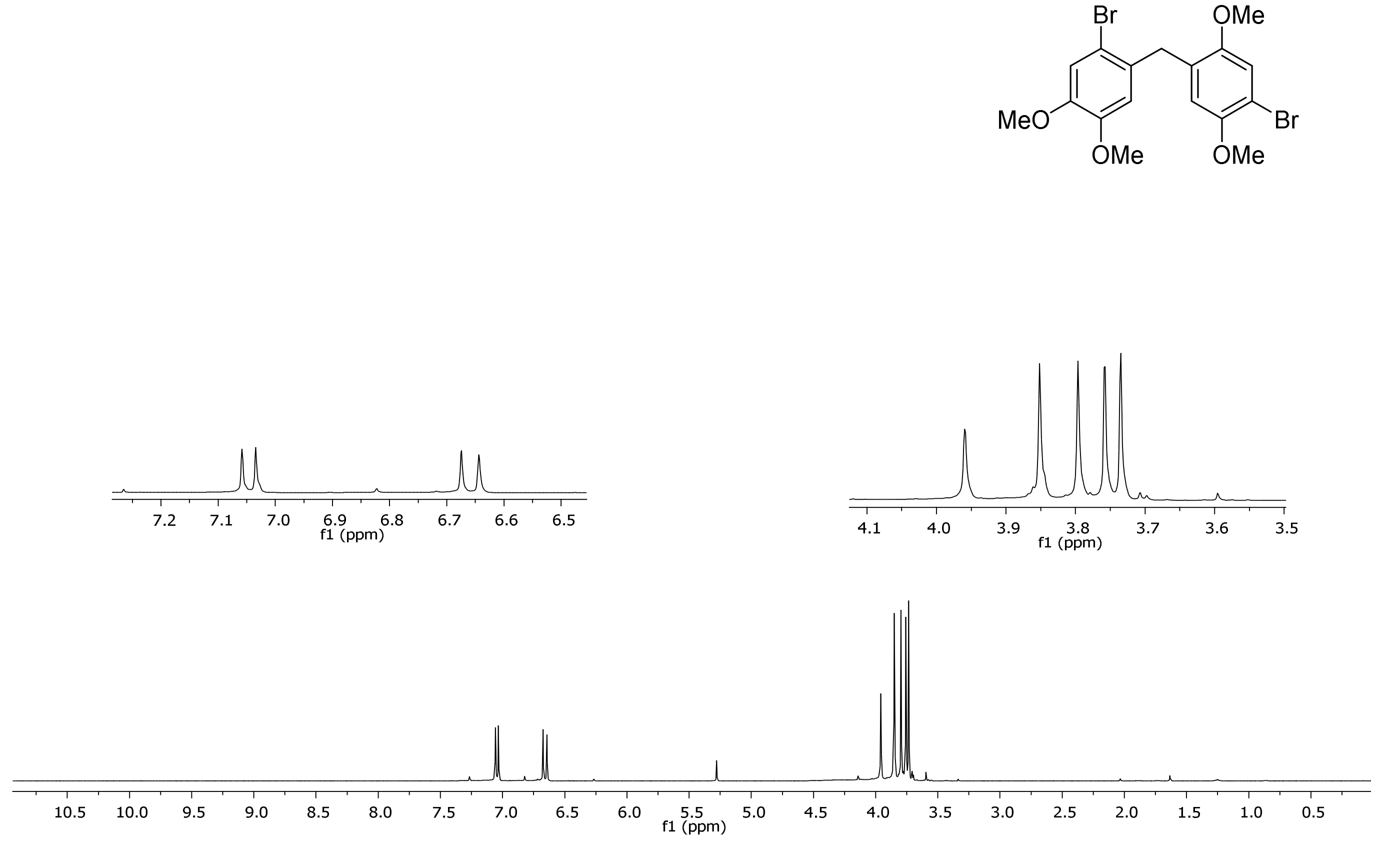

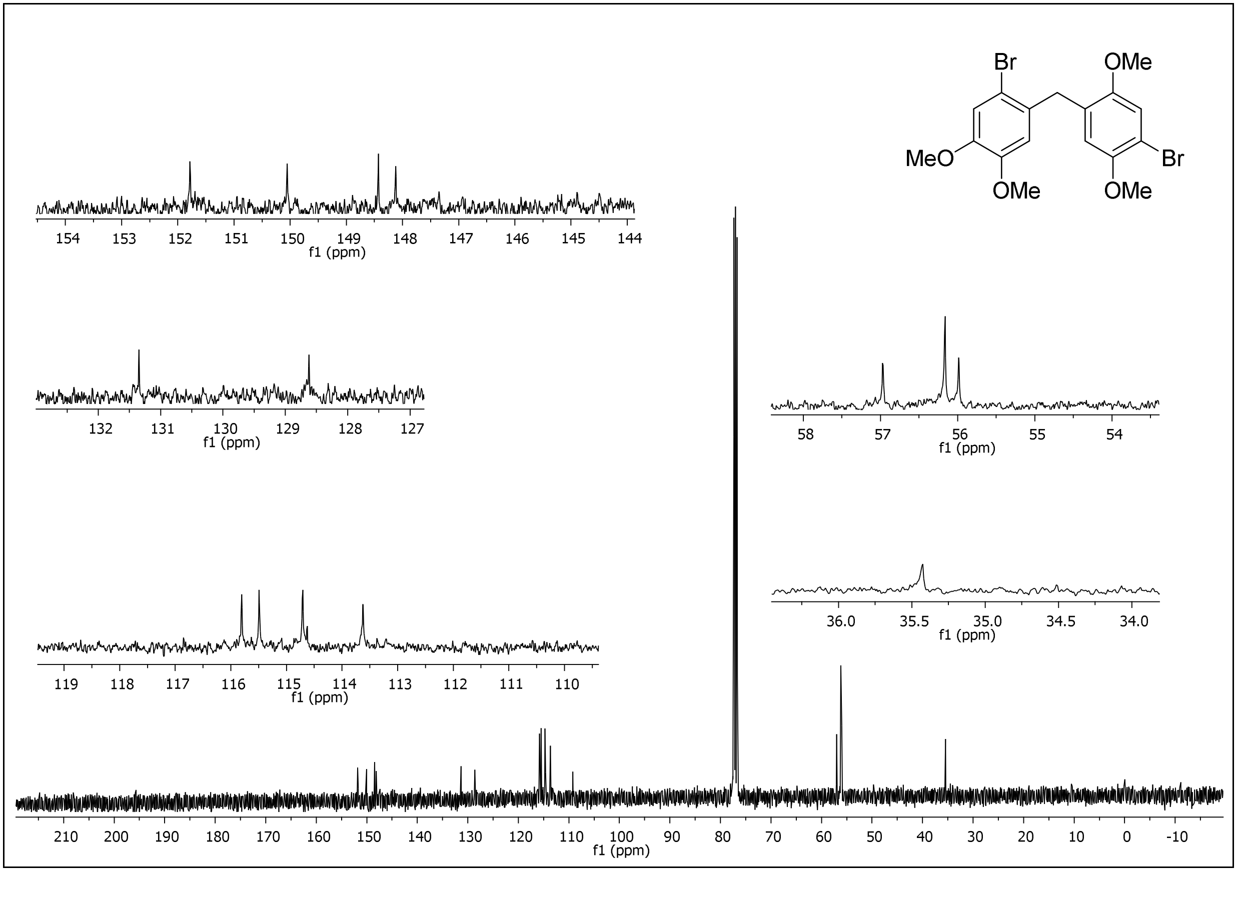

^{1}H-NMR and ^{13}C-NMR of compound 16 ($CDCl_3$)

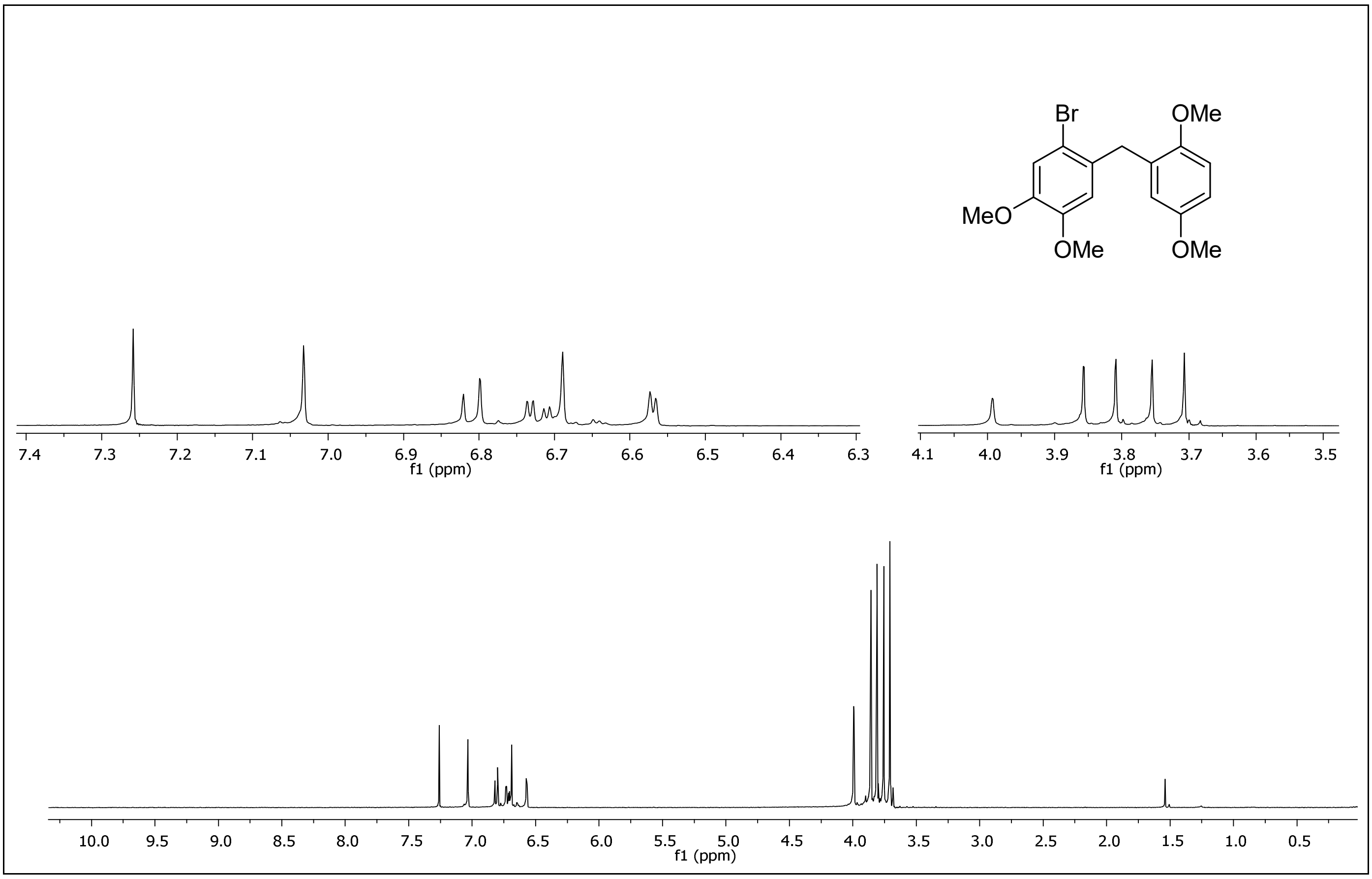

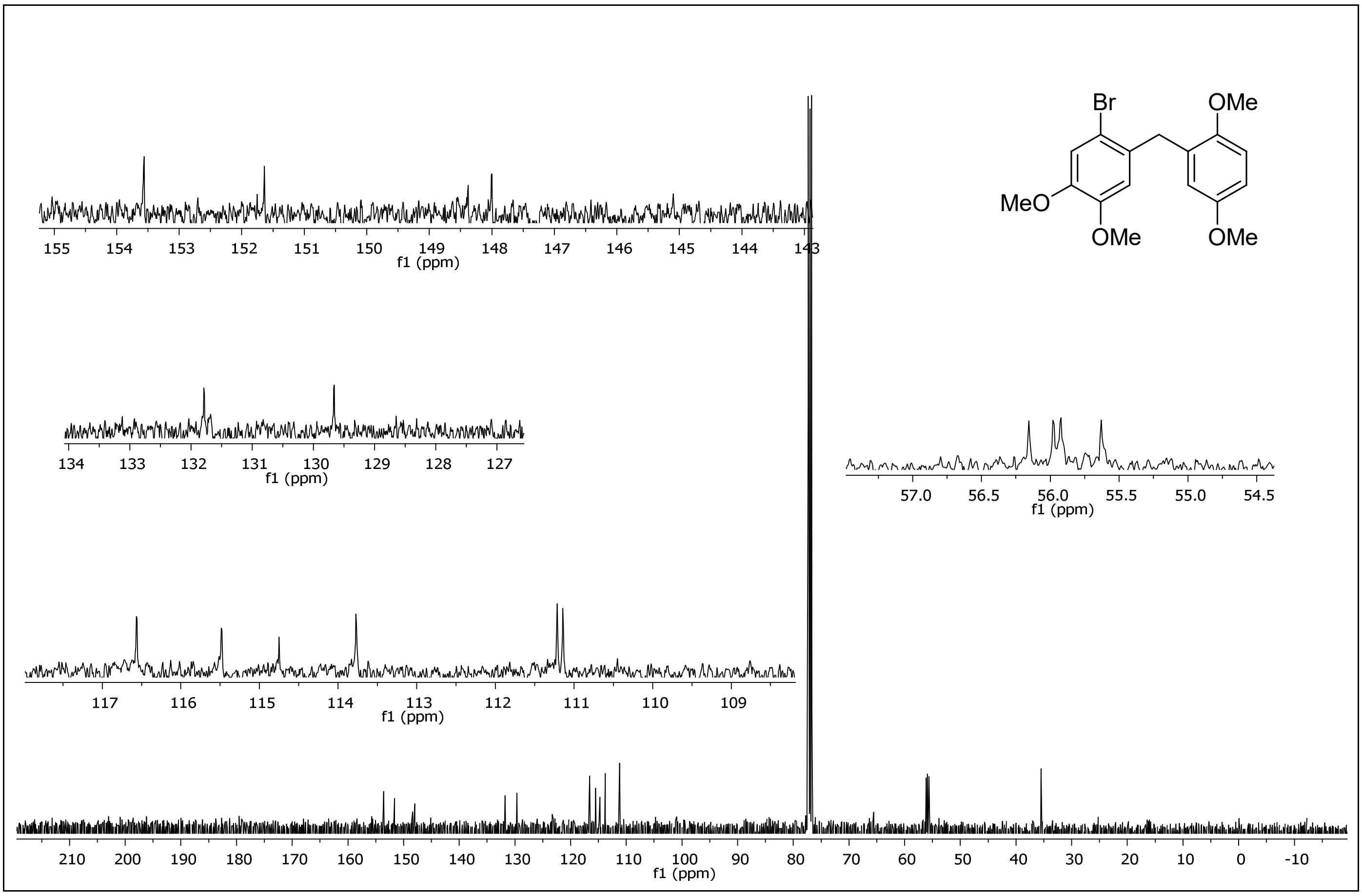

^{1}H-NMR and ^{13}C-NMR of compound 17($CDCl_3$)

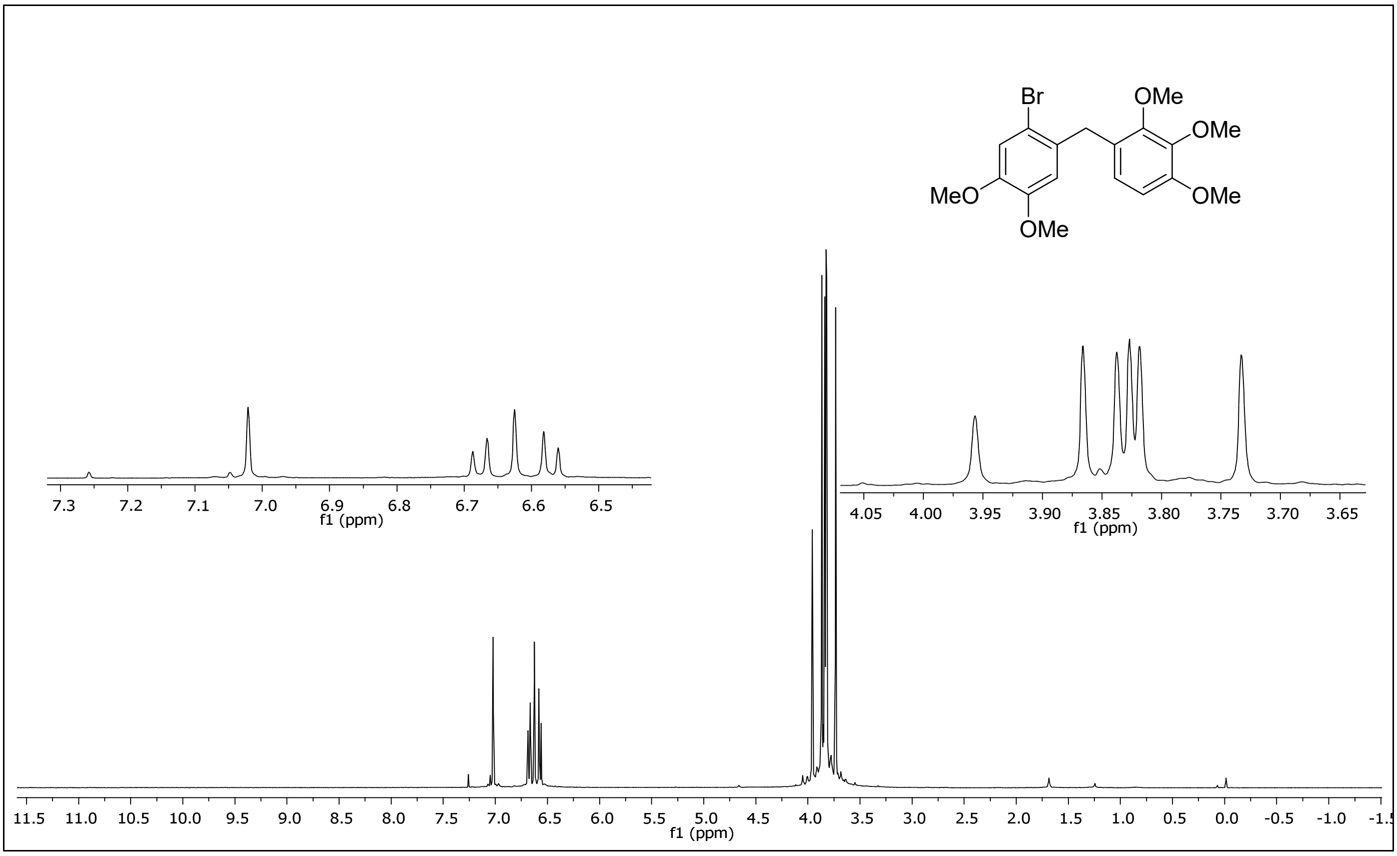

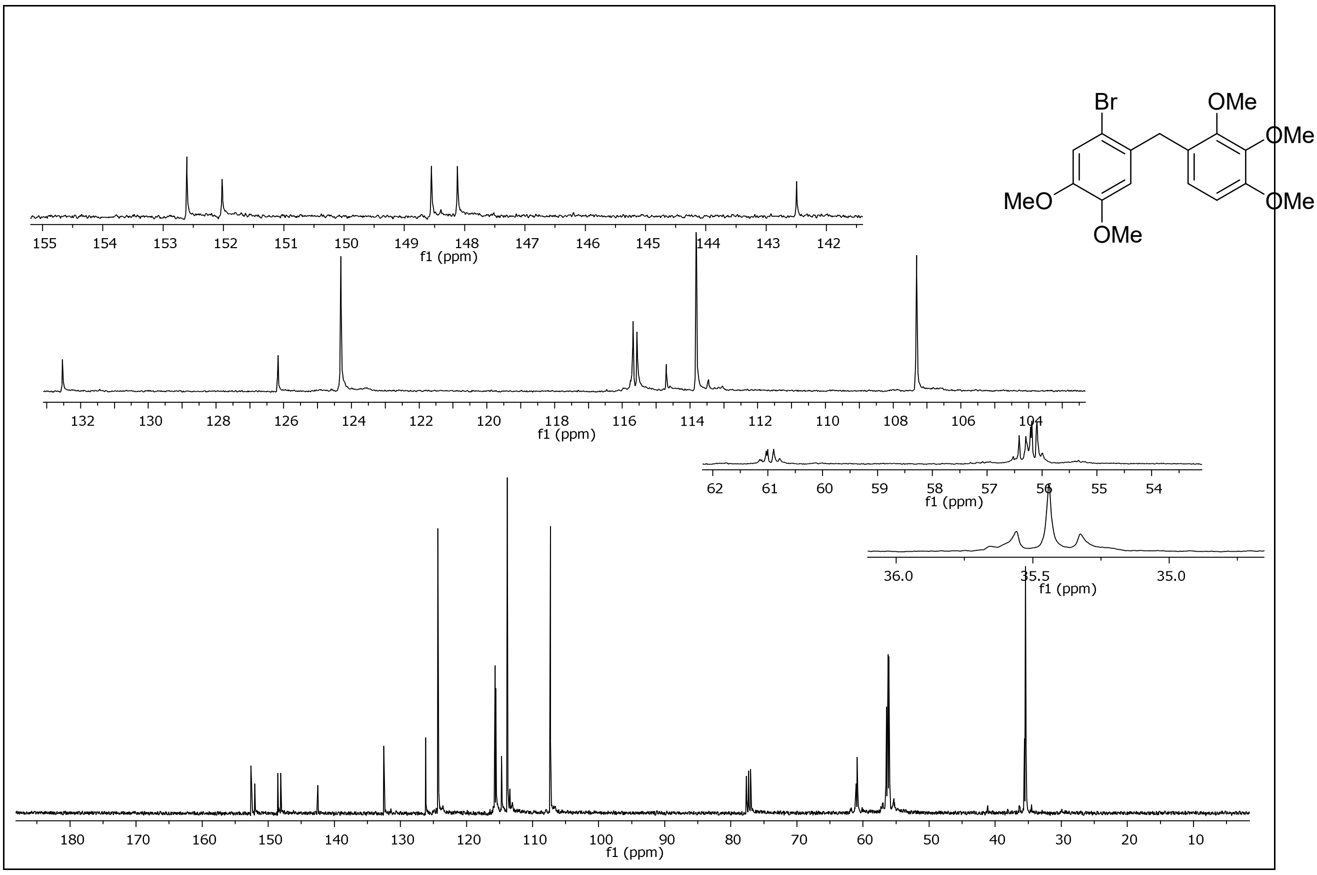

^{1}H-NMR and ^{13}C-NMR of compound 18 (CD_3COCD_3)

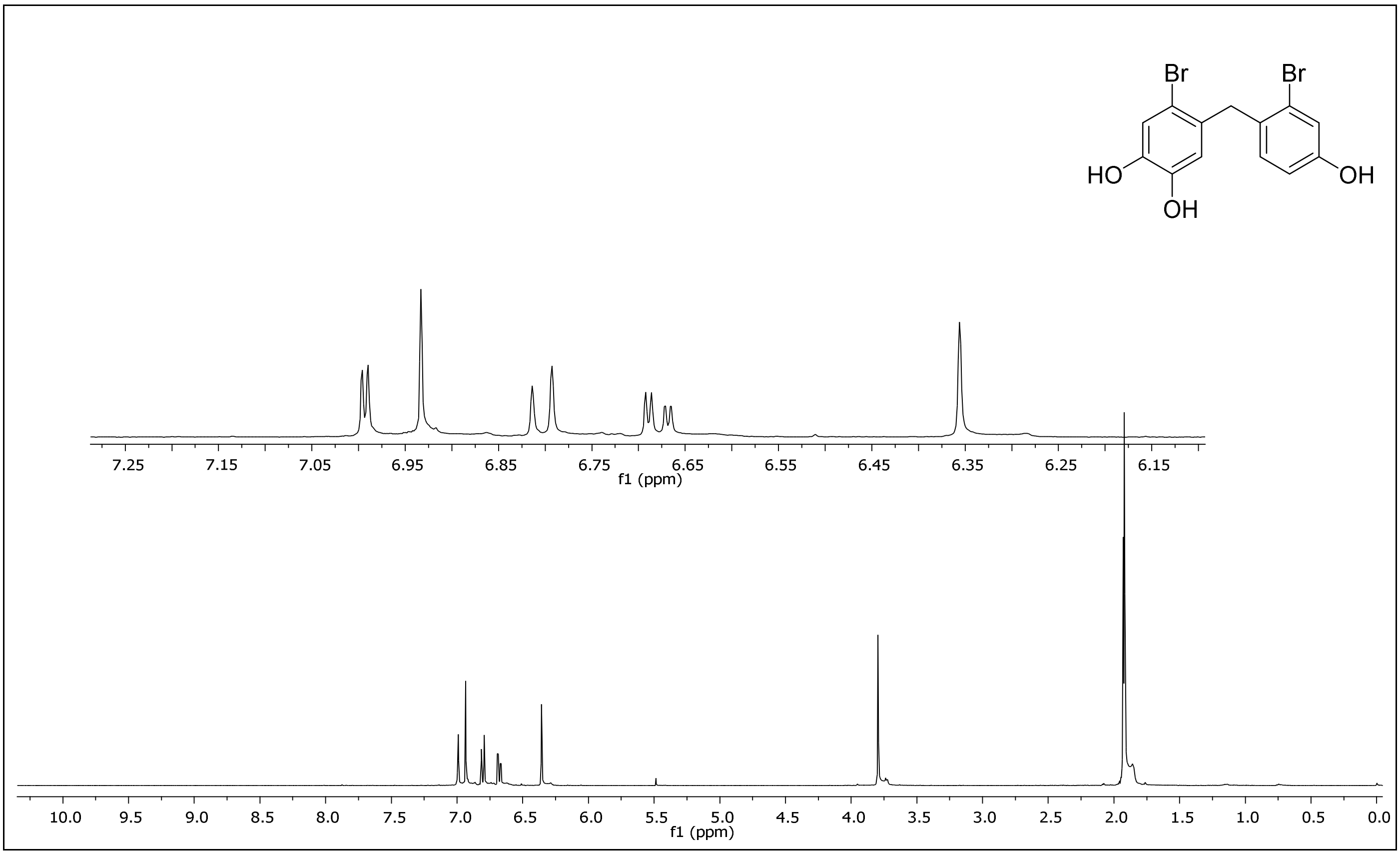

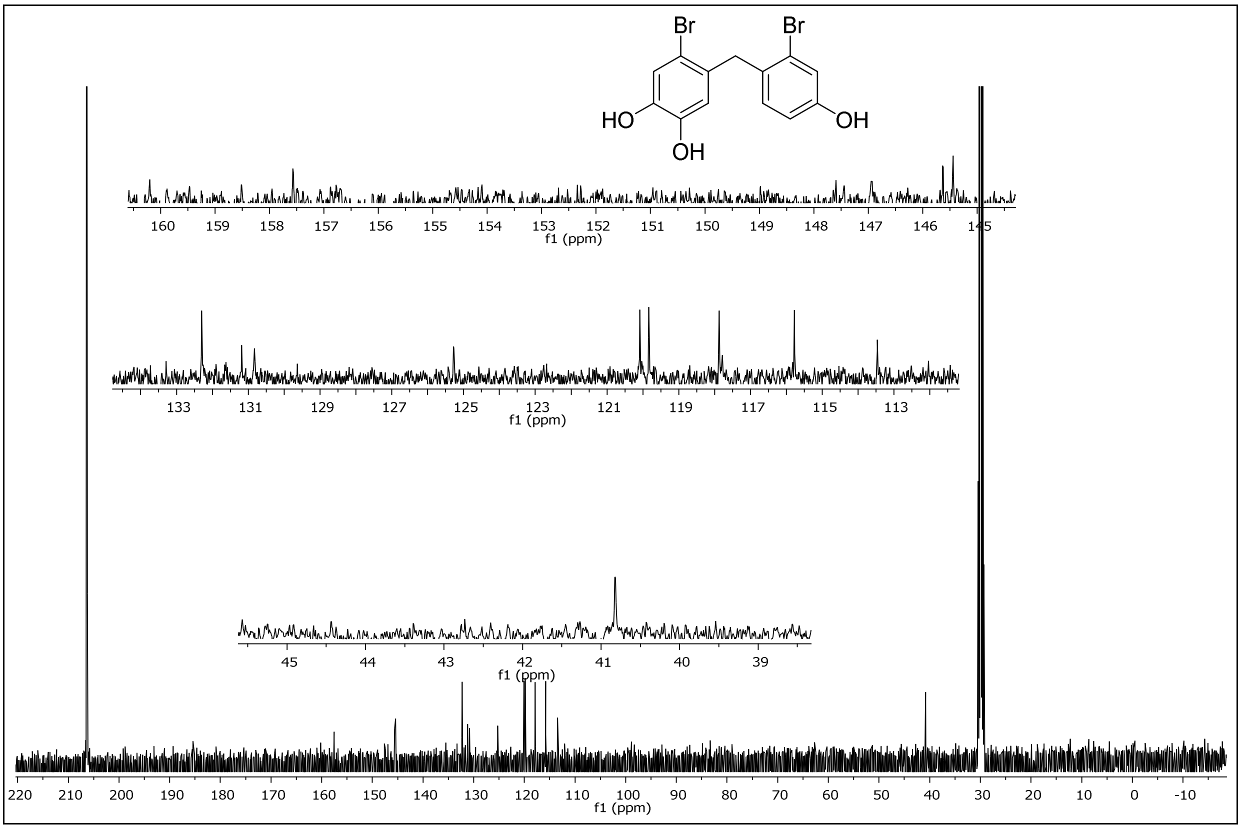

^{1}H-NMR and ^{13}C-NMR of compound 19 (CD_3COCD_3)

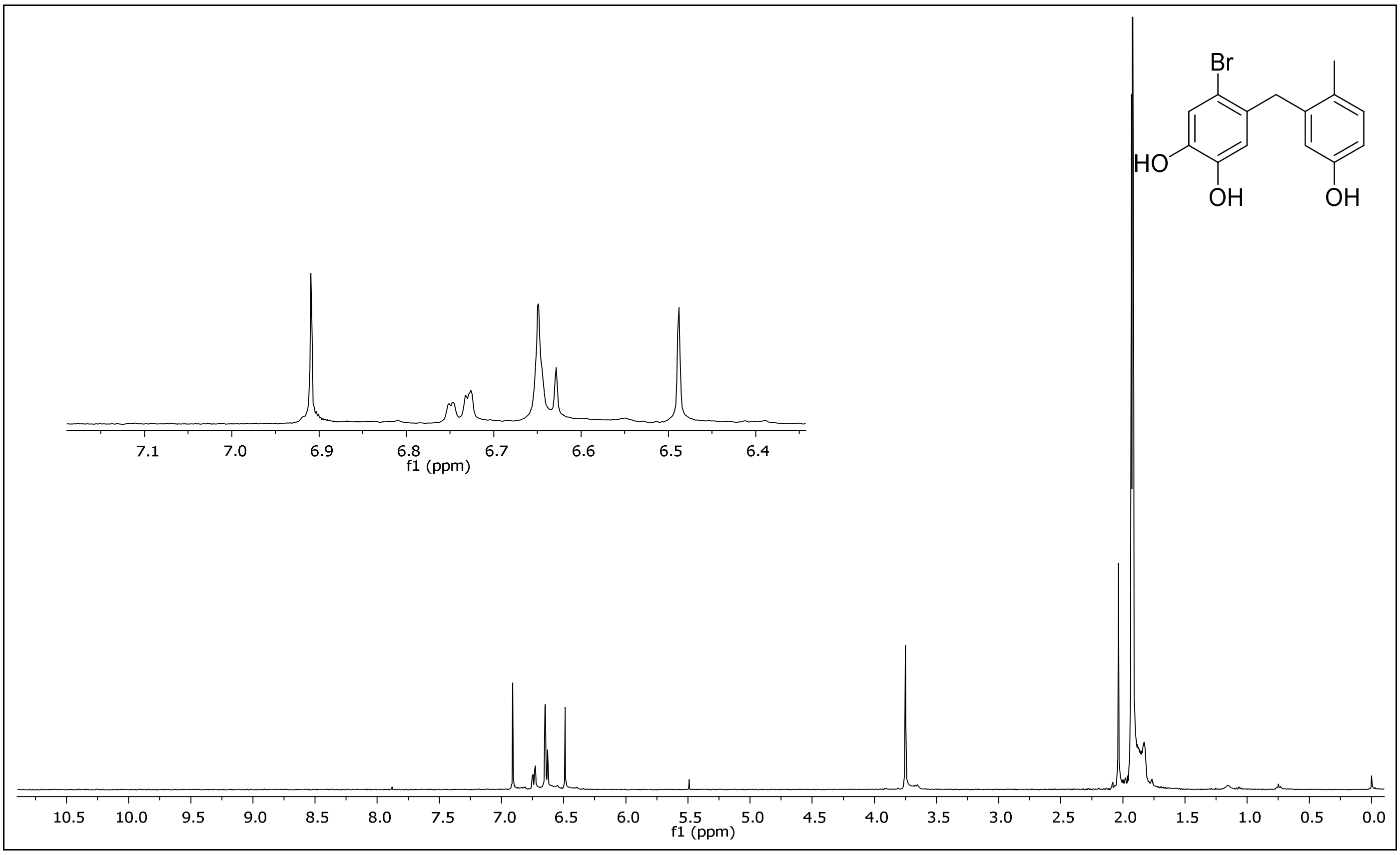

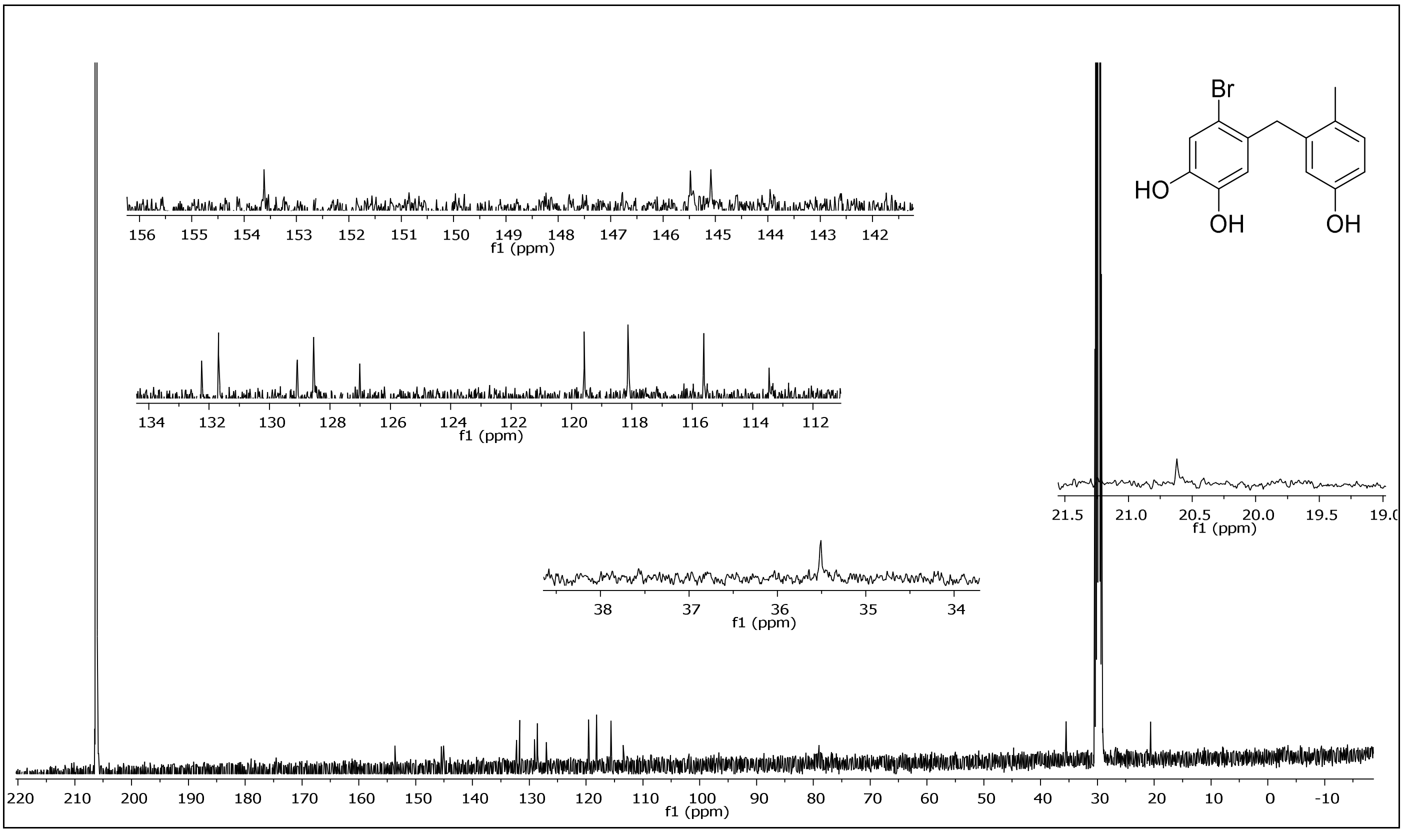

^{1}H-NMR and ^{13}C-NMR of compound 20 (CD_3COCD_3)

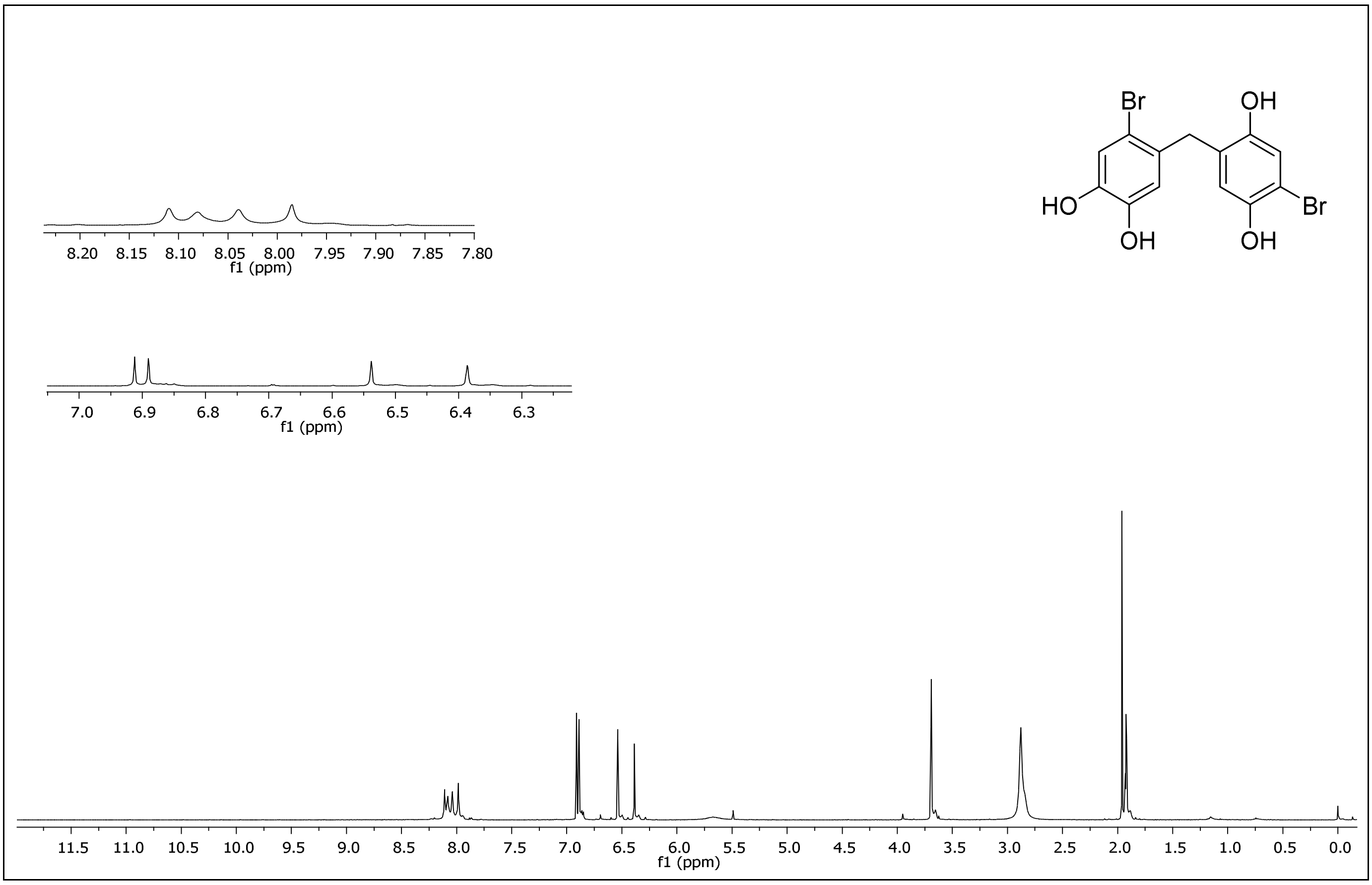

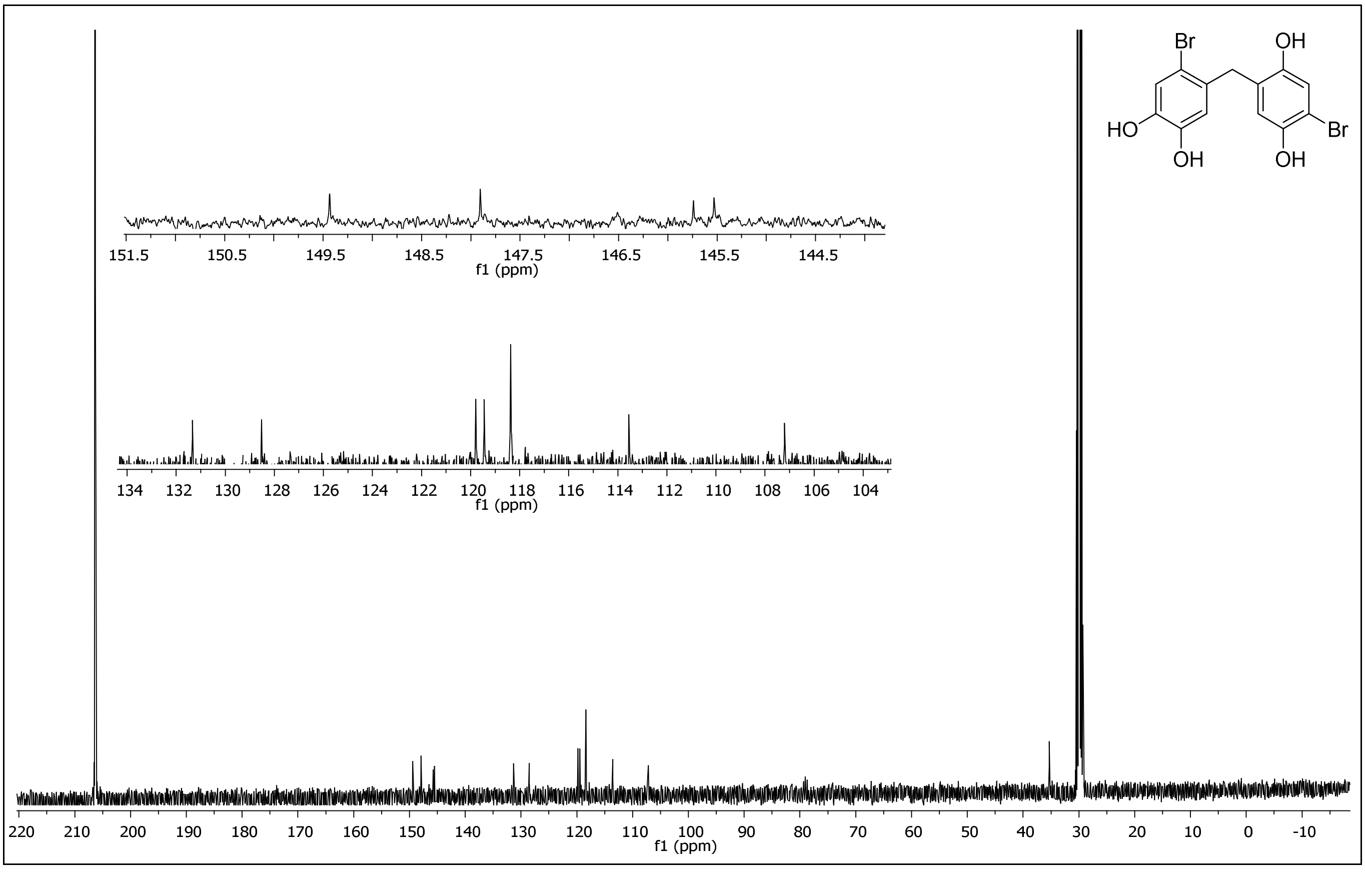

^{1}H-NMR and ^{13}C-NMR of compound 21 (CD_3COCD_3)

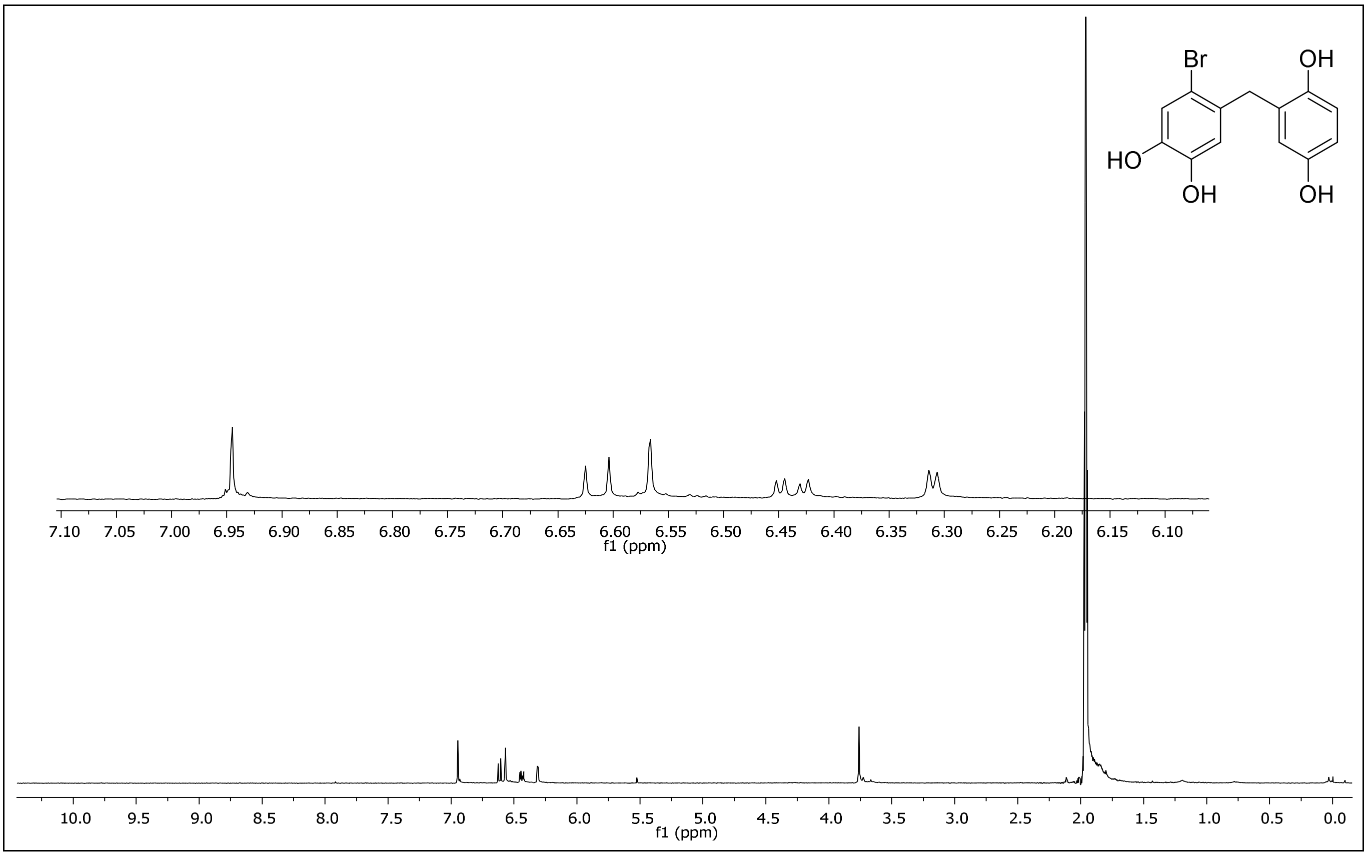

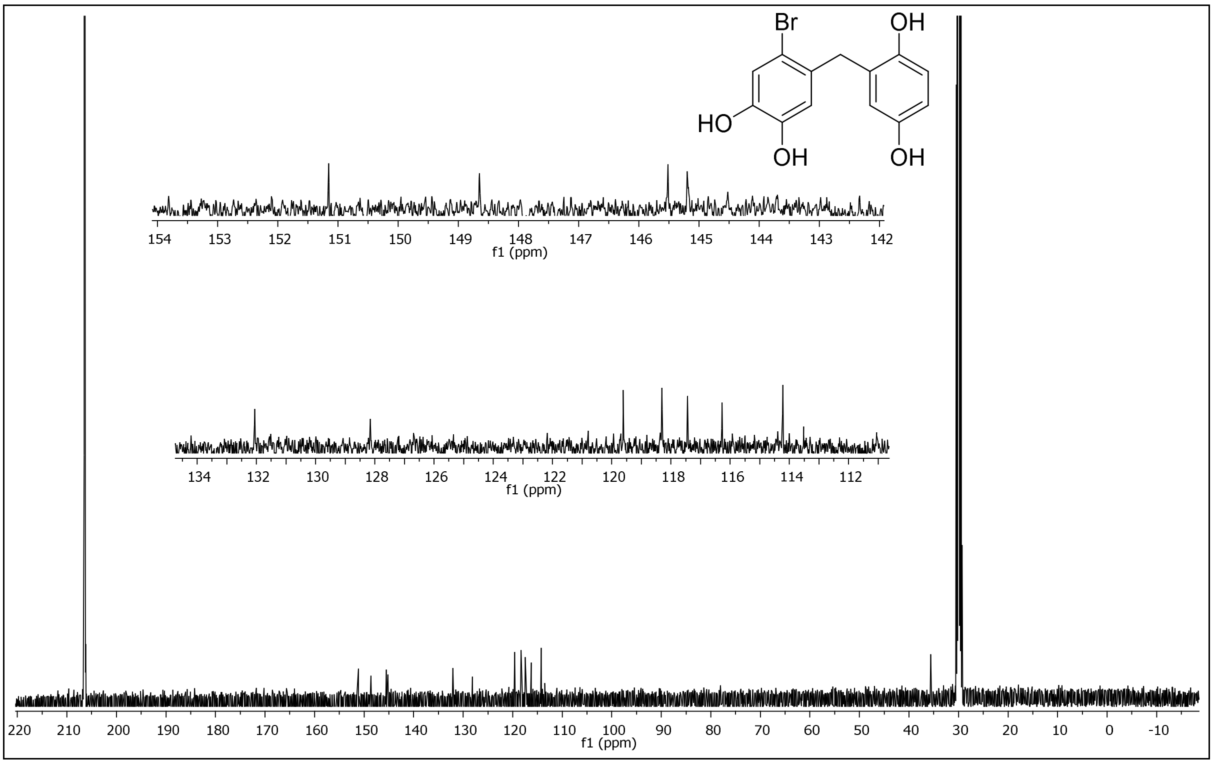

IR spectra

1-bromo-2-(2-bromo-4-methoxybenzyl)-4,5-dimethoxybenzene(13)

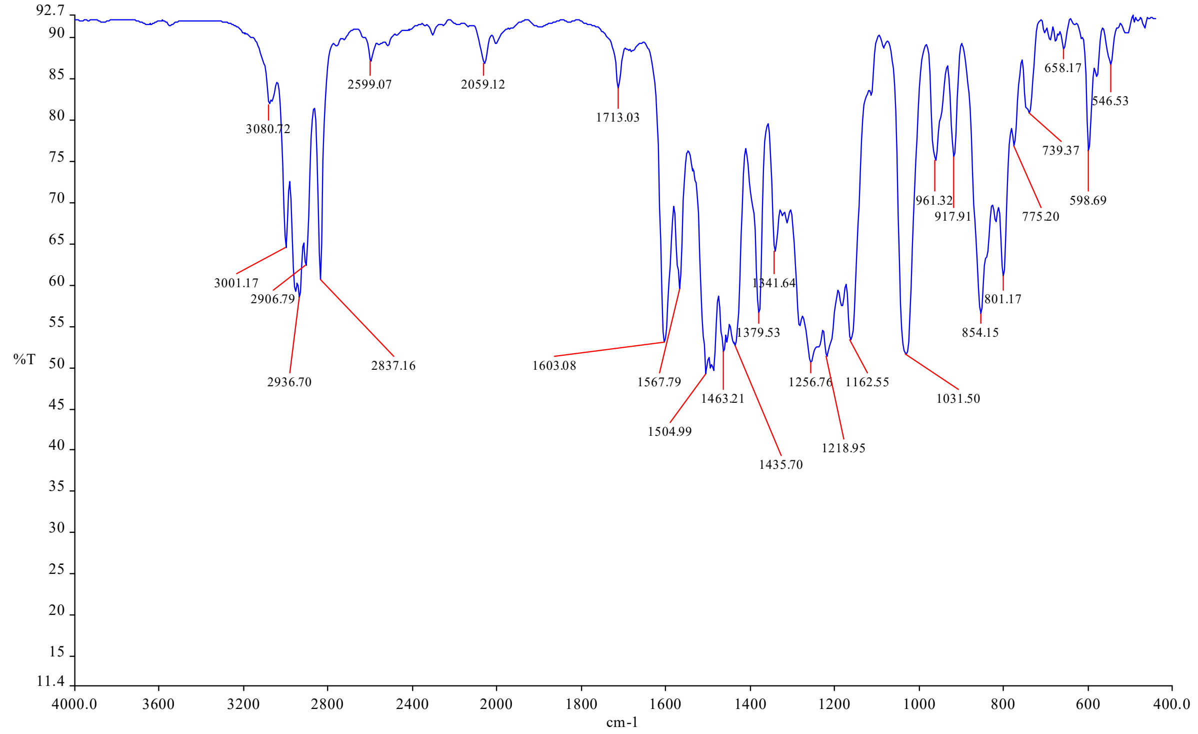

1-bromo-4,5-dimethoxy-2-(5-methoxy-2-methylbenzyl)benzene (14)

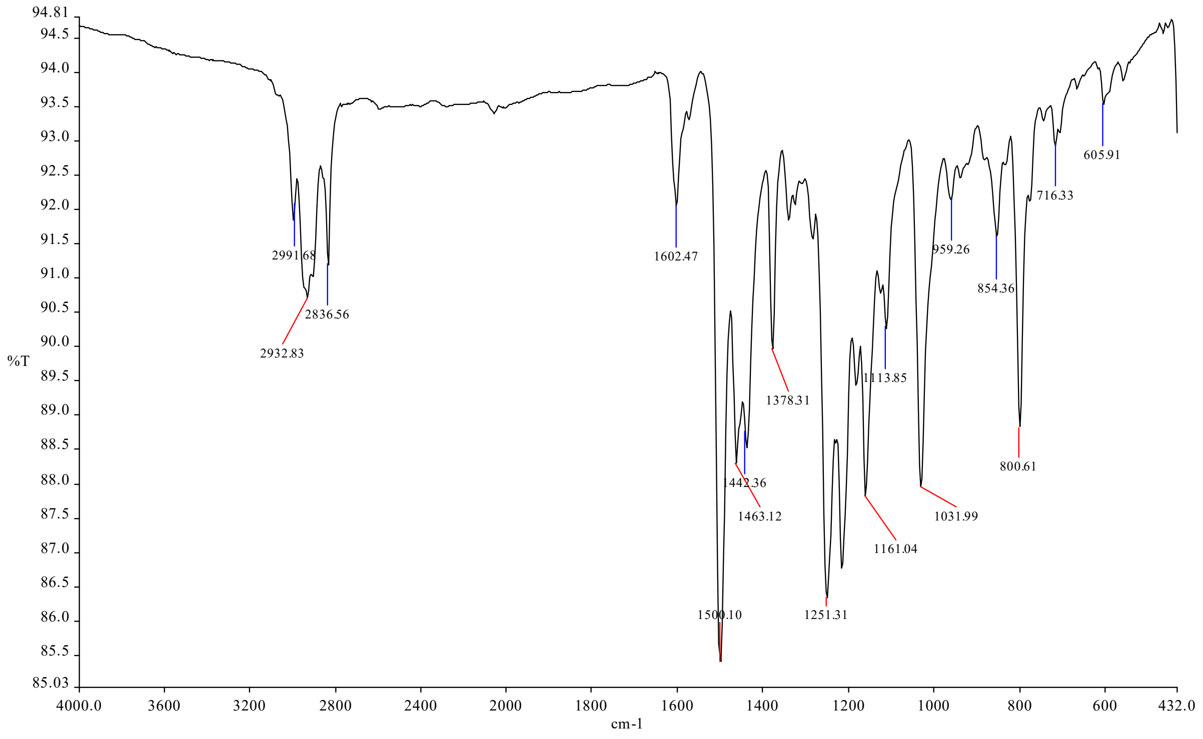

1-bromo-2-(4-bromo-2,5-dimethoxybenzyl)-4,5-dimethoxybenzene (15)

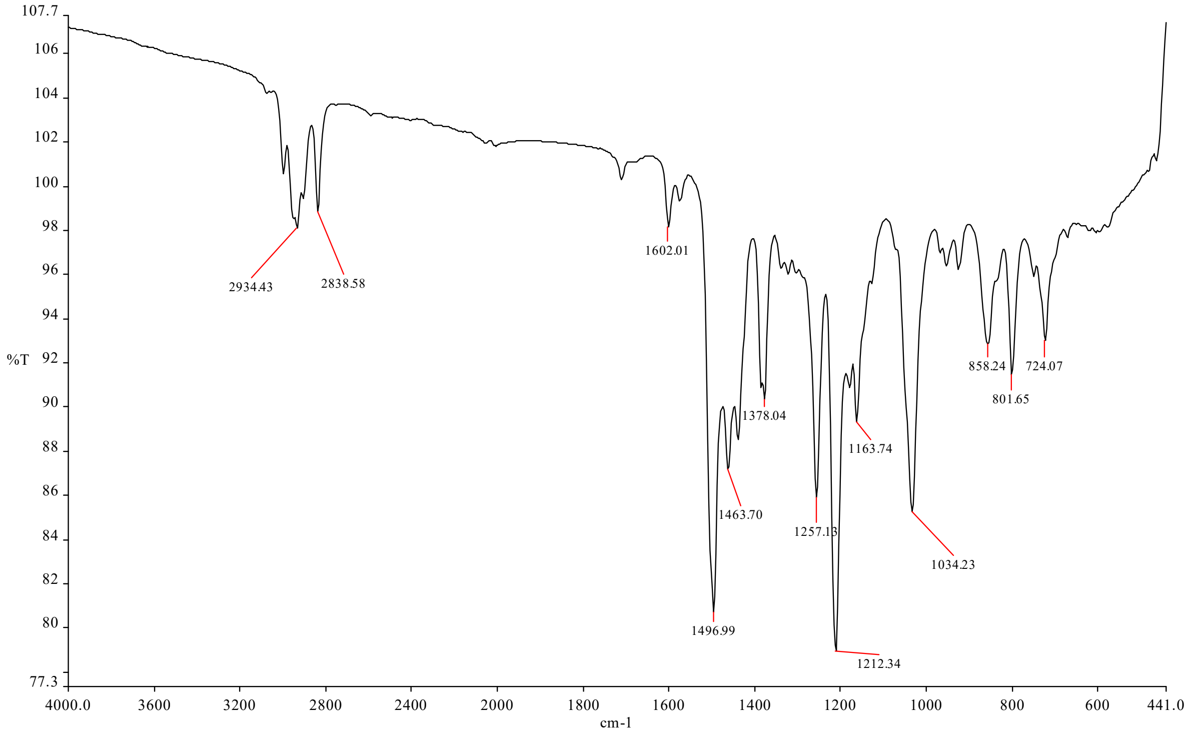

1-bromo-2-(2,5-dimethoxybenzyl)-4,5-dimethoxybenzene (16)

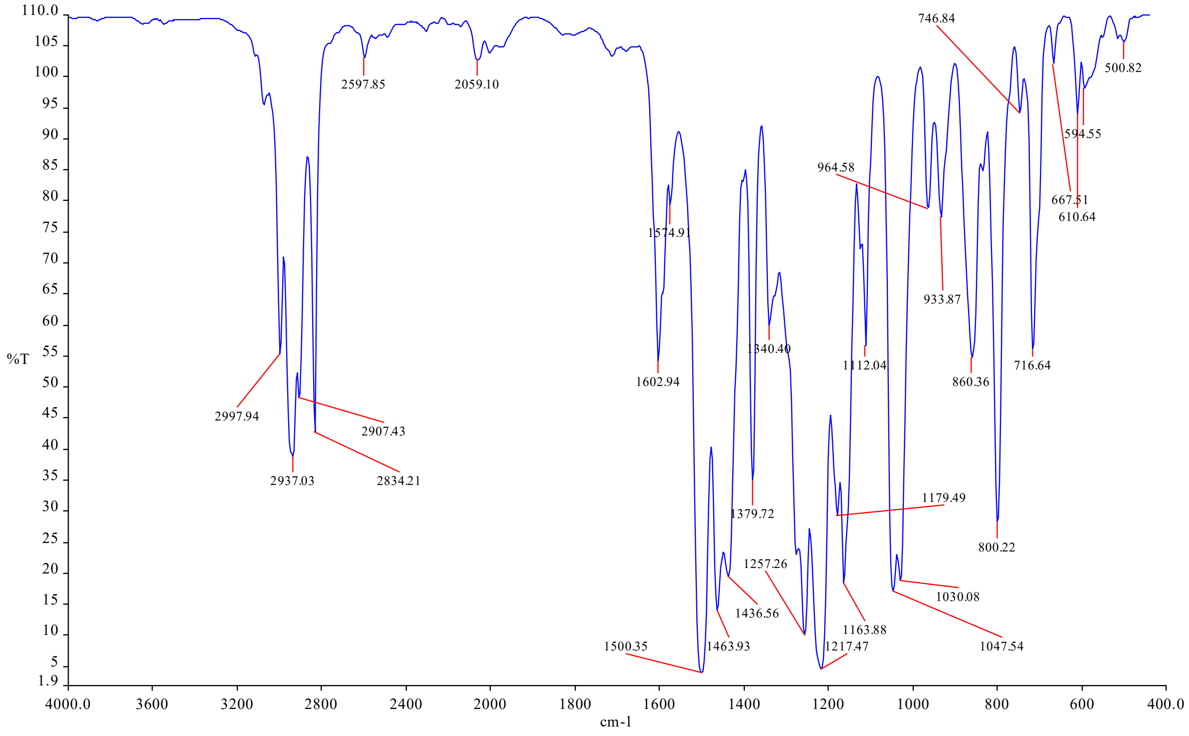

1-bromo-2-(2,5-dimethoxybenzyl)-4,5-dimethoxybenzene (17)

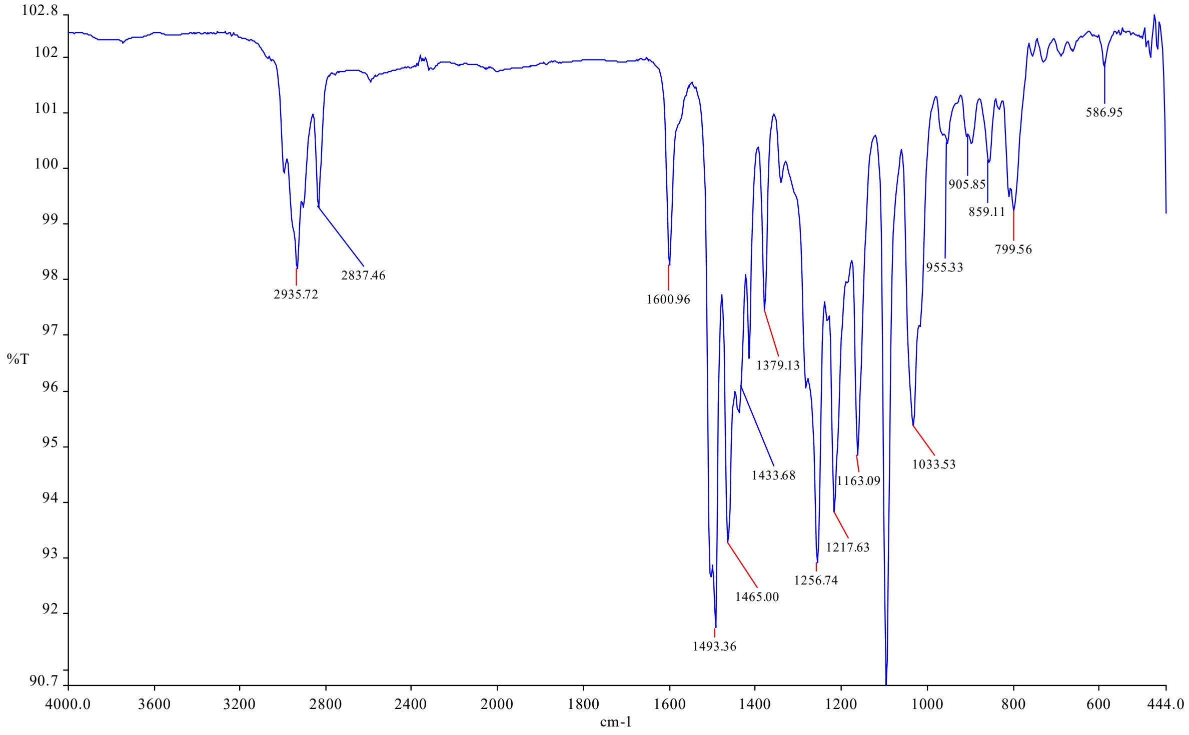

4-bromo-5-(2-bromo-4-hydroxybenzyl)benzene-1,2-diol (18)

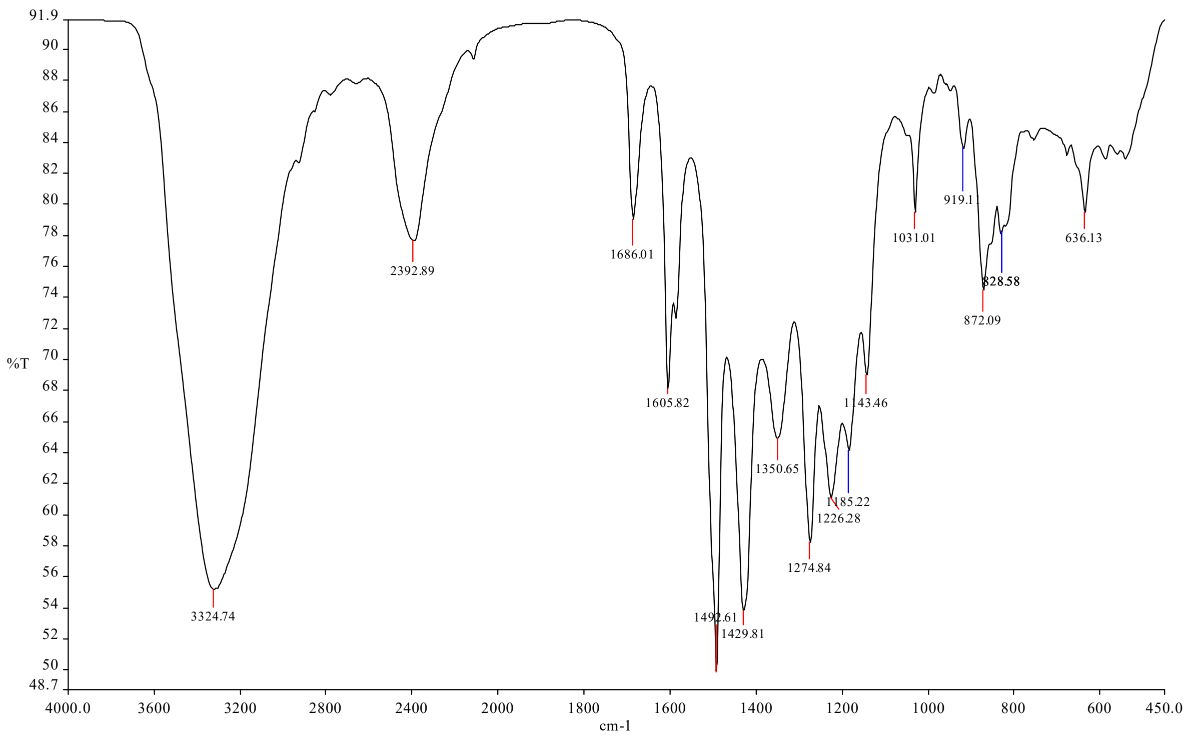

4-bromo-5-(2-bromo-4-hydroxybenzyl)benzene-1,2-diol (19)

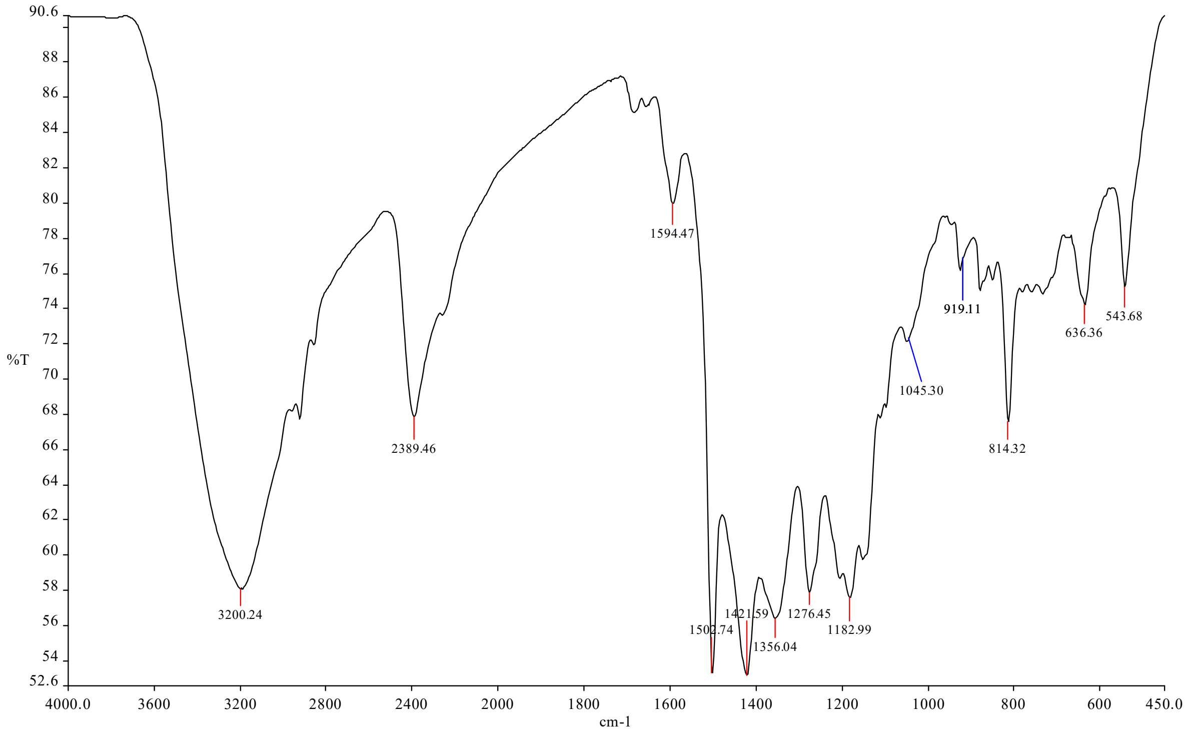

4-bromo-5-(4-bromo-2,5-dihydroxybenzyl)benzene-1,2-diol (20)

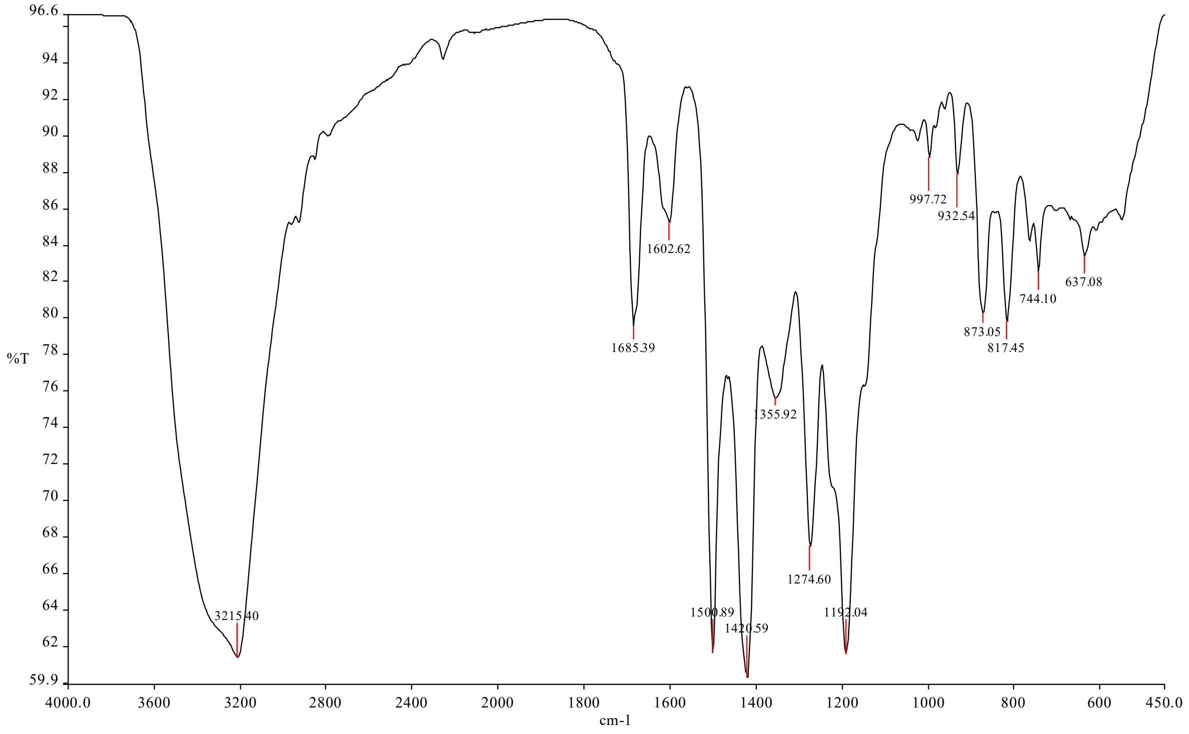

4-bromo-5-(2,5-dihydroxybenzyl)benzene-1,2-diol (21)

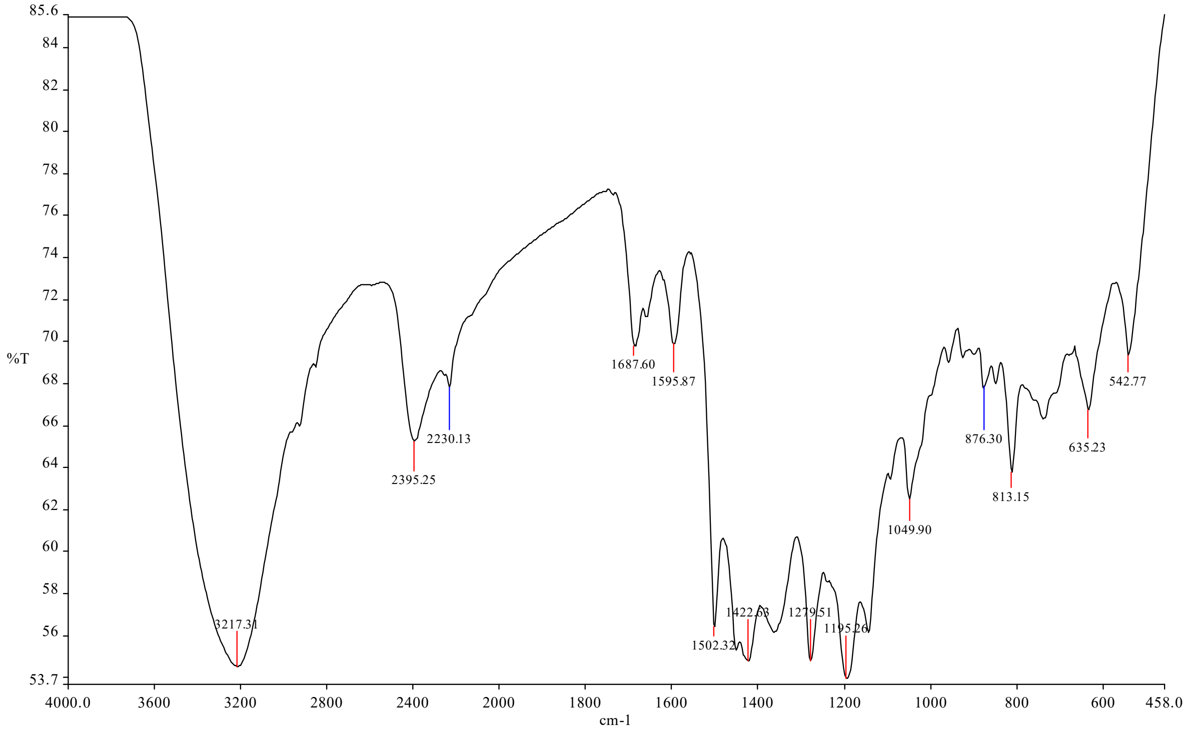

Supplement: Supplementary file 1 [file molecules-27-07426-s001.zip › molecules-1951437-supplementary.pdf]
